# Nuclear Ribosomal ITS Functional Paralogs Resolve the Phylogenetic Relationships of a Late-Miocene Radiation Cycad *Cycas* (Cycadaceae)

**DOI:** 10.1371/journal.pone.0117971

**Published:** 2015-01-30

**Authors:** Long-Qian Xiao, Michael Möller

**Affiliations:** 1 Key Laboratory of Tropical Forest Ecology, Xishuangbanna Tropical Botanical Garden, Chinese Academy of Sciences, Mengla, Yunnan, China; 2 Science Division, Royal Botanic Garden Edinburgh, Edinburg, Scotland, United Kingdom; University of Florida, UNITED STATES

## Abstract

*Cycas* is the most widespread and diverse genus among the ancient cycads, but the extant species could be the product of late Miocene rapid radiations. Taxonomic treatments to date for this genus are quite controversial, which makes it difficult to elucidate its evolutionary history. We cloned 161 genomic ITS sequences from 31 species representing all sections of *Cycas*. The divergent ITS paralogs were examined within each species and identified as putative pseudogenes, recombinants and functional paralogs. Functional paralogs were used to reconstruct phylogenetic relationships with pseudogene sequences as molecular outgroups, since an unambiguous ITS sequence alignment with their closest relatives, the Zamiaceae, is unachievable. A fully resolved and highly supported tree topology was obtained at the section level, with two major clades including six minor clades. The results fully supported the classification scheme proposed by Hill (2004) at the section level, with the minor clades representing his six sections. The two major clades could be recognised as two subgenera. The obtained pattern of phylogenetic relationships, combined with the different seed dispersal capabilities and paleogeography, allowed us to propose a late Miocene rapid radiation of *Cycas* that might have been promoted by vicariant events associated with the complex topography and orogeny of South China and adjacent regions. In contrast, transoceanic dispersals might have played an important role in the rapid diversification of sect. *Cycas*, whose members have evolved a spongy layer in their seeds aiding water dispersals.

## Introduction

Cycadales, commonly known as cycads, represent the oldest living seed plants that have been in existence for more than 200 million years [1]. They exhibit some morphological characteristics intermediate between ferns and angiosperms, such as dichotomous branching; pollen tubes that release motile sperm; and ovules, borne on the margins of leaf-like megasporophylls, that contain a large, free-nuclear megagametophytic stage [2–5]. These characteristics imply that cycads constitute a key group in plant evolution, and Norstog (2003) referred to them as the “Rosetta Stone” in understanding the origin and early evolution of seed plants [6].

Nowadays, cycads are restricted to tropical and subtropical regions occurring in highly isolated populations, in spite of an almost worldwide distribution in the Mesozoic era [7]. The majority of species are either threatened, critically endangered, or on the brink of extinction [8], and all species have been listed in the *Convention on International Trade in Endangered Species of Wild Fauna and Flora* (CITES). Cycads comprise three living families, Cycadaceae, Stangeriaceae, and Zamiaceae [9], though the monophyly of Stangeriaceae has been challenged [10–12]. Within Cycadaceae, the genus *Cycas* is currently recognized as the sole genus with 107 species [13], though a separate Asian genus *Epicycas* was once described [14]. Within *Cycas*, taxonomic relationships remain quite controversial, and multiple competing classification schemes have been proposed [15–18]. All these taxonomic treatments place importance on the shape of the megasporophyll, but differ in the degree to which the megasporophyll is emphasized compared to other characteristics of the leaf, stem, and ovule [17]. Despite the prehistoric appearance of *Cycas*, molecular dating analyses pointed to a rapid speciation for the extant species in the Miocene [19–22]. This suggested that taxonomic confusion within this genus could result from rapid speciation, rather than the conservative morphological evolution. Lineages experiencing rapid radiations represent a challenge for reconstructing their molecular systematics, because of the difficulty of resolving short branches from successive cladogenic events, retention of ancestral polymorphisms or the occurrence of hybridization [23]. Indeed, there are still no independent molecular phylogenies addressing clearly the debated delimitations and evolutionary relationships of intra-generic taxonomic units of *Cycas*. This could have been hampered by the low phylogenetic differentiation in DNA sequences [19, 20, 24], and the confounding effect of spurious phylogenetic relationships of nuclear ribosomal DNA (nrDNA) ITS pseudogenes [25] that have been inadvertently included in previous analyses [12]. It is challenging but necessary to evaluate independent molecular sequence regions to reconstruct a phylogenetic framework within *Cycas*, and to compare the resulting in evolutionary relationships with morphological classifications.

The nr DNA region consists of several hundreds to thousands tandem paralog repeats, located at one or several loci in each haploid genome [26]; the nrDNA copies evolve in a concerted manner under molecular processes of unequal crossing over and gene conversion. As part of the nrDNA unit, the ITS region has the advantages of rapid concerted evolution among the paralogs, fast evolutionary rate, and suitable length and availability of universal PCR primers [27]. They have thus become the most popular molecular marker in the nuclear genome for species-level phylogenetic inference of plant groups. In *Cycas*, however, the high degree of intra-individual polymorphism detected among ITS paralogs suggested a non-concerted evolution of rDNA. These divergent paralogs included not only functional genes but also putative pseudogenes and recombinants [25]. Among the divergent ITS paralogs, recombinants will obscure real phylogenetic relationships that may exist [28]. However, the phylogenetic significance of the pseudogenes and functional paralogs remain ambiguous. Some argue that pseudogenes evolve independently under essentially neutral conditions and might be helpful for studying evolutionary genetics [29]. Functional paralogs on the other hand, are subject to selection for function and compensatory mutations [30], which may result in homoplasy, and thereby confound phylogenetic signal [31]. The ITS pseudogenes in *Pyrus* [32] and *Corymbia* [33] provided two empirical examples whereby the phylogenetic relationships were resolved well. Others argue that pseudogenes diverge free from evolutionary constraint, which prompts concerns about sequence alignment and long-branch attraction issues [34]. Therefore, we cannot necessarily rely on these paradoxical theoretical assumptions to predict the phylogenetic implications of pseudogenes and require empirical data in order to ascertain the information content in ITS pseudogenes and functional paralogs. In *Cycas*, our previous study on a small sample size (six species) indicated that the homoplastic mutations could have confounded the phylogenetic signal in pseudogenes. Phylogenies inferred from the functional ITS paralogs, however, showed well-supported species-specific clades, which bodes well for studies on a larger sample size to provide further insights into the evolution on this genus [25].

Although *Cycas* are charismatic plants with a high profile for plant conservation, many phylogenetic relationships remain unresolved. Understanding the evolutionary relationships within this group is essential for inferring the putative causes and routes of diversification within a historic and geographic context. In the present study, we cloned and sequenced the nrDNA ITS regions from 31 species, covering all morphological and geographical diversity of *Cycas*. We further examined the patterns of non-concerted evolution of the nrDNA ITS arrays, identified functional copies and used these to clarify the phylogenetic relationships and trace the evolutionary history of the genus. We further linked these patterns with the different seed dispersal capabilities, and paleo-geography, and tried to elucidate the main forces behind the evolutionary processes in *Cycas*.

## Materials and Methods

### Ethics statement

All necessary permits were obtained for the sample collection in the two botanical gardens.

### Taxon sampling

Thirty-one species (one individual per species) were sampled from cultivated plants at the Fairy Lake Botanical Garden, Shenzhen, and Xishuangbanna Tropical Botanical Garden, CAS. Only old trees, originally collected directly in the field, were selected to avoid potential problems of ‘genetic pollution’ from hybridization among the cultivated individuals. This sampling accounts for about one-third of all described species in *Cycas*, which has covered the main distribution areas and the taxonomic and morphological diversity of the genus. Representatives of all recognized sections and subsections in the classification proposed by Hill (2004) [17] were included, except for subsection *Lindstromiae* in section *Indosinenses*, which includes only one species, *C. lindstromii*, from Vietnam. Section affiliations, information on origin, and herbarium vouchers are given in S1 Table. Voucher specimens are deposited in the Xishuangbanna Tropical Botanical Garden herbarium (HITBC), China.

### Molecular methods and sequence analyses

Total genomic DNA was extracted from silica gel dried leaves following a CTAB protocol [35]. The methods previously described were used to acquire cloned ITS sequences and to identify pseudogenes, recombinants and functional paralogs among the intra-genomic divergent ITS copies, involving a range of comparisons of sequence characteristics of functional cDNA ITS copies, including sequence length, substitution variation, GC content, secondary structure stability, the presence of a conserved motif in the 5.8S gene, 5′–GAATTGCAGAATCC–3′ [36], and evolutionary rates [25]. GENECONV tests were performed using the substitution model for putative recombinant detection, as implemented in the Recombination Detection Program [37]. For each species, cloning and sequencing were carried out until at least two functional sequences were obtained. These sequences have been deposited in GenBank, and the accession numbers of previously and newly acquired cloned nrDNA sequences for all species considered in this study are given in S2 Table. For incorporating indel characters into the analyses, the simple indel coding (SIC) method [38] was applied using SeqState version 1.2 [39]. The resulting indel matrix was combined with the nucleotide sequence matrix. MrModeltest v. 2.2 [40] and the Akaike information criterion were used to select a GTR+G model of nucleotide substitution for the analysis.

### Phylogenetic analyses

To evaluate the relationships between pseudogenes and functional paralogs, a maximum likelihood (ML) phylogenetic analysis was conducted using RAxML [41]. The GTR+G model of nucleotide substitution was used for DNA data, and the binary indel characters treated as a separate partition using a BINGAMMA evolutionary model. Support for the estimated tree was assessed using 1000 bootstrap replicates. Because of the large evolutionary gap between *Cycas* and its closest sister, the family Zamiaceae [10–12], the divergence of ITS sequences is too great to permit confident alignment between *Cycas* and Zamiaceae (as a potential outgroup). Thus, in theses analyses, only *Cycas* samples were included and the phylogenetic tree displayed unrooted.

### Rooting of the phylogeny

Paralogs can serve as better outgroups than sister species especially for groups without closely related extant taxa [26, 33, 42]. For the reason given above regarding outgroup and ingroup alignment, we rooted the phylogeny on all pseudogenes, since they all clustered together irrespective of the species they were obtained from, and separate from their corresponding functional paralogs in the gene tree based on all paralogs. Among the pseudogenes, *C. armstrongii*_5, *C. circinalis*_11, *C. javana*_5, and *C. media*_9 were close to the functional paralogs in the ML gene tree based on all paralogs. To check for a possible effect of long-branch attraction in the phylogeny topology, we also used only these four pseudogenes as outgroup samples in the phylogenetic analysis. We also checked all combination of alternative combinations as outgroup samples, with the aim to test whether the selection of pseudogenes as outgroup samples had an effect on the resulting topology of the functional paralogs.

Since RAxML analysis cannot collapse the tree nodes with zero or near zero lengths, and may leads to spurious resolution characterized by the combination of high resolution and little to no bootstrap support. To overcome this shortcoming, less resolved topologies need to be evaluated in addition to completely resolved topologies [43]. In this study, the phylogenetic trees were also constructed using Bayesian inferences (BI) with MrBayes [44]. Four Markov chains starting with a random tree were run simultaneously for 2 million generations, sampling every 100 generations. The nucleotide substitution model used was GTR+G as above, and the binary characters were included as a separate binary restriction data partition, using the command “coding = variable” under “lset”. As a convergence diagnostic, the average standard deviation of split frequencies had fallen below 0.0058. After discarding the first 25% of tree topologies as burn-in, topologies were summarized as a majority-rule consensus tree. This analysis was run twice to confirm convergence between independent runs. In this analysis, each of the four selected pseudogenes was tried to use as outgroup.

### Biogeographical analysis

We used a Bayesian Binary MCMC (BBM) method implemented in Reconstruct Ancestral States in Phylogenetics (RASP) v3.0 beta [45]) to reconstruct the possible ancestral ranges at nodes on the phylogenetic trees. The current distribution areas of *Cycas* species (S1 Table) were obtained from Hill (1998) [46] and coded as follows: (A) South China, plus Taiwan-Ryukyu Archipelago, and Palawan islands. The former was connected to the South China mainland via land bridges during Quaternary glaciations [47]; the latter drifted away from the margin of the South China continental crust toward the Sulu Sea in the mid Miocene [48]. (B) Indochina, which is separated from South China by the Red River Fault (RRF) [49], corresponding to the Tanakai-Kaiyong Line, a strong biogeographic divide [50, 51]. (C) Islands of Southeast Asia, plus the Malay Peninsula, the Indian subcontinent, East Africa and North Australia, where the *Cycas* species have a natural distribution in near-coastal regions, rather than inland as in the aforementioned two areas. Although the components of this area have different paleogeographic origins [52], these *Cycas* species have the potential for oceanic dispersal between these components (see discussion). To account for uncertainties in the phylogeny, we used 20,001 trees from MrBayes’ output, discarded the first 5,000 trees (representing the burn-in trees from the Bayesian analysis), and ran the BBM analysis on the remained 15,001 trees. The outgroup pseudogenes were removed from the analyses. The four MCMC chains with a temperature of 0.1 were run simultaneously for two million generations, sampled every 100 generations, and the first 5,000 samples were discarded as burn-in. State estimation was run under the F81 + G model for the BBM analysis. The maximum number of areas for this analysis was kept to 3.

## Results

### ITS sequences

**Fig 1:**
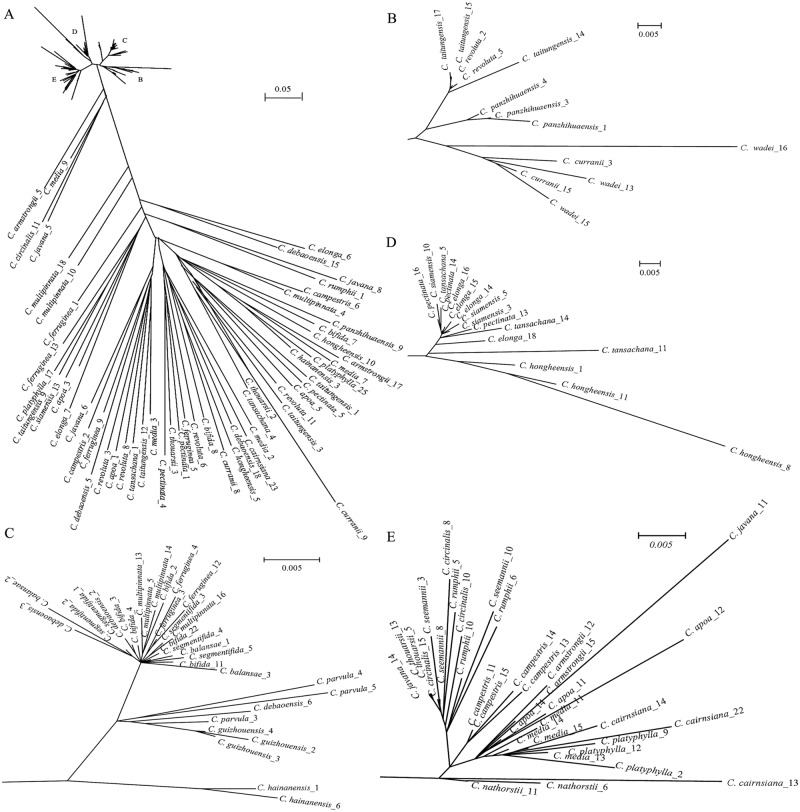
Unrooted phylogram of the RAxML maximum likelihood analysis. Results of the RAxML maximum likelihood analysis of 149 ITS paralogs obtained from thirty-one species of *Cycas* (excluding 12 putative recombinants). A) Unrooted phylogram of all samples; B-E) magnified views of clades indicated in A).

A total of 161 distinct genomic ITS sequences were obtained from the 31 *Cycas* species. The functional analyses to distinguish between functional ITS paralogs and pseudogenes / recombinants showed that among these, 94 were functional sequences and 66 were pseudogenes. The putative pseudogenes were generally distinguished by their significantly lower GC contents, and the lower secondary structure stability of 5.8S, and lack of a conserved seed plant specific 14-bp motif in 5.8S (S2 Table). Among the pseudogenes, 12 were recombinants (S2 Table), which displayed sharp discontinuities in the patterns of sequence similarity and substitution pattern among the ITS paralogs. Recombinants were *C. bifida*_1, *C. ferruginea*_6, *C. nathorstii*_3 and 12, *C. panzhihuaensis*_1, *C. pectinata*_7, *C. siamensis*_15, *C. taitungensis*_16 and 18, *C. thouarsii*_8 and 21, and *C. wadei*_7. The unrooted ITS ML tree (*ln*L = -30469.093188) showed that the functional and pseudogene paralogs each were monophyletic, with the pseudogenes possessing extremely long terminal branches and short internal branches (Fig. 1).

### Phylogenetic analyses

**Fig 2:**
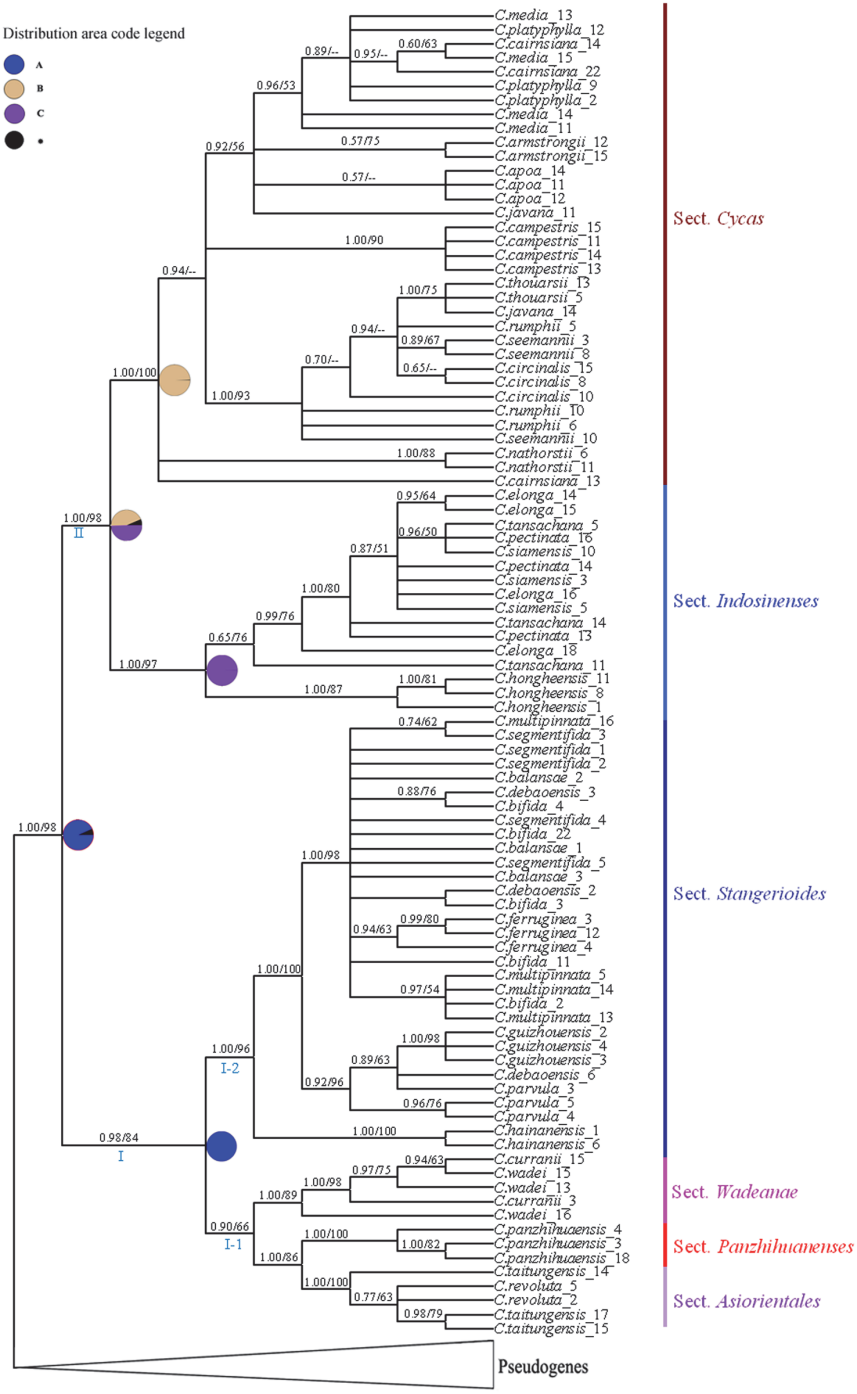
Rooted phylogram of the Bayesian analysis. Bayesian 50% majority-rule consensus cladogram showing evolutionary relationships of 94 functional ITS paralogs and 4 pseudogenes (*C. armstrongii*_5, *C. media*_9, *C. javana*_5, and *C. circinalis*_11). Here *C. circinalis*_11 was chosen as an outgroup sample. Bayesian posterior probabilities (BPP), and bootstrap support values of ML (MLBS) analyses are shown above branches, respectively (BPP/ MLBS, dashes indicate BPP or MLBS values < 50%). Ancestral distribution inferred from Bayesian analysis with RASP were also labeled on the cladogram; pie charts beside internal branches represented ancestral distributions as probabilities coded as follows: A (South China), B (Indochina), C (Islands of Southeast Asia).

The aligned functional ITS sequences matrix contained 1118 characters, of which 591 were constant and 527 were variable. The topologies from the BI (Fig. 2) and ML (*ln*L = -9163.063561) analyses depicted nearly identical phylogenetic relationships with the only differences that the resolved branches with low supports in the ML tree were collapsed into polytomy in the BI tree. The utilisation of all pseudogenes as outgroup and the approach to use various selections of pseudogenes as outgroup samples resulted in the same topologies (data not shown). In the phylogenetic analyses, the functional ITS sequences were monophyletic (BPP = 1.00, MLBS = 98), with two main clades (clade I: BPP = 0.98, MLBS = 84; clade II: BPP = 1.00, MLBS = 98) (Fig. 2). The species-specific functional ITS paralogs were paraphyletic and polyphyletic. However, the ITS sequences of the species clustered in six clades, each representing a section recognized by Hill (2004) [17]. Clade I was composed of species belonging to sects *Asiorientales* (BPP = 1.00, MLBS = 100), *Wadeanae* (BPP = 1.00, MLBS = 89), *Stangerioides* (BPP = 1.00, MLBS = 96), and sequences of the monotypic *Panzhihuaenses* (BPP = 1.00, MLBS = 100). Within Clade I, sects *Asiorientales*, *Panzhihuaenses* and *Wadeanae* clustered together and formed subclade I-I (BPP = 90, MLBS = 66), sister to sect. *Stangerioides*. Within this subclade, sects *Asiorientales* and *Panzhihuaenses* were sister (BPP = 1.00, MLBS = 86), and together, were sister to the Palawan sect. *Wadeanae*. The Clade II samples shared a 14-bp deletion near the ITS1 5′-end, except for two functional paralogs in *C. hongheensis* (clone No. 8 and 11). Clade II consisted of two well-supported subclades, corresponding to sects *Indosinenses* (BPP = 1.00, MLBS = 97) and *Cycas* (BPP = 1.00, MLBS = 100).

### Biogeography analysis

The biogeographical analysis strongly favored a scenario of a South China origin for the extant *Cycas* species (93.95%), with an early dispersal to Indochina, and suggested a vicariant event between South China and Indochina (46.11%). The ancestral distribution of the ancestor of clade II (sects *Indosinenses* and *Cycas*) was inferred to be most likely the Indochina region (49.09%) with a series of later dispersals to the islands of Southeast Asia, the Indian subcontinent, East Africa and North Australia (Fig. 2).

## Discussion

### Pattern of divergent ITS paralogs

As expected, the divergent nrDNA ITS sequences isolated from 31 species of *Cycas* could be categorized as pseudogenes, recombinants or functional paralogs, and the functional and pseudogene paralogs each formed a clade in the all-ITS-paralog-inclusive phylogenetic tree (Fig. 1). Within their clades, the pseudogenes clustered randomly across species and sections, whereas the functional ITS paralogs formed six clades corresponding to the sections of Hill [17]. However, the functional paralogs mostly did not form species-specific clades, with only a few exceptions. This might suggest that, at the species level, the diversity of the functional ITS paralogs predates speciation events and that incomplete lineage sorting events caused the observed non-monophyletic pattern of the species copies [53]. In some cases, the differences between the species-copies were small, one or a few substitutions, and these could potentially have originated from PCR or sequencing errors. This might need to be investigated in a larger, more detailed study. However, our findings already indicate that when more than one functional sequence from a species is included in species-level phylogenetic studies, the potential flaws resulting from incorrect assumptions of orthology can be avoided. It is interesting that all functional paralogs obtained from several species (such as *C. panzhihuaensis*, *C. hongheensis*,*C. guizhouensis*, *C. ferruginea* and *C. hainanensis*) were monophyletic and formed well-supported clades. This pattern suggested that these species could have experienced intensive founder or bottleneck events, which would have accelerated coalescence of all functional paralogs to their most recent common ancestor. Of course, the relatively small sample size of functional ITS paralogs per species and species sample size of one used here, could also have led to these patterns by chance.

### Outgroup role for ITS pseudogenes

Outgroup samples are a prerequisite for rooting a phylogenetic tree to determine the evolutionary topology of the ingroup taxa. However, this can present a problem for phylogenetically isolated groups where the sister group is too distantly related to the ingroup taxa to align the sequences [54], and particularly when there is relatively little variation among the in-group taxa, the outgroup method could be challenging and the results misleading [55]. In these cases, a solution is to use a molecular outgroup rather than an organism outgroup [56]. *Cycas* is such a case. It is an isolated lineage only distantly related to other cycads [10–12], and the extant species show relatively low genetic differentiation among species [24, 57, 58]. Identification of divergent paralogs will provide molecular outgroup opportunities for phylogenetically isolated taxa, when the branching of the pseudogene from the functional paralogs is older than the common ancestor of all the functional paralogs [33, 42, 56]. The reciprocal monophyly in the phylogenetic tree between functional paralogs and pseudogenes suggested that the pseudogenes could be used as outgroups when inferring evolutionary species relationships within *Cycas* from the functional paralogs. As an alternative, we investigated the possibility of using pseudogenes as outgroups in *Cycas*. These were relatively easily and unambiguously aligned with the functional copies when included as outgroups and root. Changing the outgroup selection in our case did not altered the resulting phylogenetic relationships. The resulting ingroup species topology was concordant with geographic distribution, and also generally compatible with those obtained from slower evolving cpDNA datasets where the outgroup method was not compromised [24]. We thus concluded that our approach appears to yield reliable phylogenetic estimates of species relationships.

### Phylogenetic relationships of *Cycas*

The monophyly of the genus *Cycas* is commonly accepted [12, 17, 18, 46], but the subdivision of the genus into subgenera and sections is greatly debated, since most classification systems were formulated on few, easily scored characters from gross morphology. *Cycas* was firstly divided into three sections using megasporophyll and ovule characters [14]. A two-section classification system was proposed based on the caudex (subterraneous *vs*. aerial) [15]. The shape of megasporophylls and seeds, and the fibrous state of the exocarp was also used to divide the genus into three groups [4]. Wang (2000) placed absolute taxonomic importance on the seed structure and proposed a classification scheme with four subgenera and seven sections [18]. Hill (2004) [17] revised his initial taxonomic treatment several times [16, 46], and finally proposed a six-section taxonomic scheme for *Cycas*. These sections consisted of sects *Asiorientales*, *Panzhihuaenses*, *Wadeanae*, *Stangerioides*, *Indosinenses* and *Cycas*.

Our work has generated the most comprehensively sampled and well-resolved molecular phylogeny for *Cycas* to date. Our phylogenetic tree showed that the species representing de Laubenfels and Adema’s (1998) [14] genus *Epicycas* (*C. multipinnata*, *C. elongata*, and *C. siamensis*) formed a polyphyletic and convergent group of species, and thus confirmed *Cycas* as the single constituent genus of Cycadaceae. Within this genus, the low-copy nuclear gene phytochrome P (*PHYP*) did not clarify the phylogenetic relationships with convincing branch support values [19]; and the cpDNA non-coding regions (combination of *trn*S-*trn*G, *psb*M-*trn*D and *trn*L-*trn*F) also provided a poor phylogenetic resolution with only three lineages resolved. These included sects *Asiorientales* and *Wadeanae*, and a clade consisting of species of sects *Cycas* and *Indosinenses* [24]. While greatly congruent in tree topology with the above analyses, the phylogenetic tree generated by the nrDNA ITS functional paralogs showed a greater resolution with much higher clade support values (Fig. 2). In this phylogenetic tree, the six lineages are strongly correlated with geography, and also corresponded exactly with the six sections recognized by Hill (2004) [17]. His taxonomic scheme was based on the most extensive studies of both herbarium and living specimens [16], and had taken a suite of morphological (reproductive and vegetative) characters as well as molecular data into consideration [17]. The sections have also been distinguished by the leaflet structure, such as, the un-lignified midrib fiber unique to section *Cycas*, and the mucilage canal present only within section *Stangerioides*. The sister relationship between section *Asiorientales* and section *Panzhihuaenses* found here was also predicted by their shared encrypted stomata and epidermal cell shape [59]. As mentioned above, the two major clades could be characterised by a 14-bp indel near the 5′-end of ITS 1. The exception for two cloned functional paralogs in *C. hongheensis* could be a sign of ancient gene polymorphism retention [60]. The two major clades also display significant differences in the microsporangiate sporophylls: those in Clade I are soft and lack an apparent apical spine, while those of Clade II are hard and have a distinct apical spine [17, 46]. Therefore, these two clades could be proposed as two subgenera of *Cycas*: subgenus *Panzhihuaenses* Wang and subgenus *Cycas*.

### Evolutionary implications for *Cycas*

**Fig 3:**
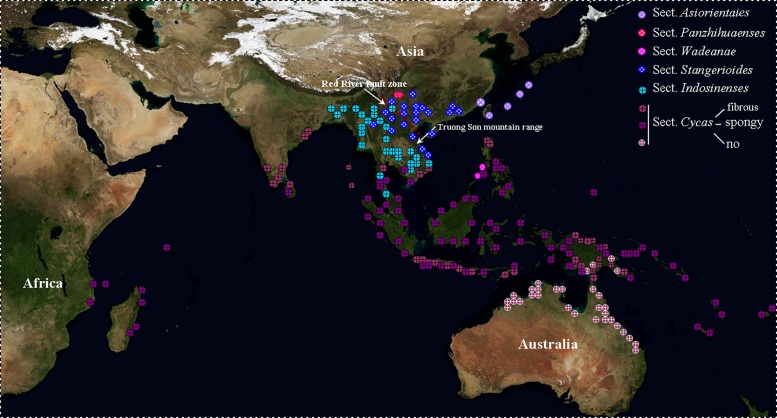
Geographic distribution of *Cycas* species (adapted from [61]). Data points were colored based on clade associations (Fig. 2).

Compared with other cycad genera restricted to a single landmass, *Cycas* has a wide distribution ranging from eastern Africa eastwards to the Pacific islands and from China and southern Japan southwards to Australia (Fig. 3) [17]. Traditionally, this paleotropical disjunctive distribution pattern was linked to Indian plate motions, which assumed that the ancestor of *Cycas* originated in East Africa during the Permian or Triassic period [62]. However, this assumption is obviously contradicted with findings of the phylogenetic patterns generated by the functional ITS paralogs and the above-mentioned molecular dating results [19, 20, 22].

Within *Cycas*, the only fossil, *C. fujiana*, was found in Kyushu of Japan and Northeast China at Eocene sites [63, 64]. This indicates that this genus could have a distribution extending further north than the existing species in the Eocene, the warmest period during the Tertiary. The subsequent general progressive global cooling could also have forced *Cycas* southward and the genus became extinct at high latitudes, similar to other thermophiles [65, 66]. The ancestral area reconstruction results, together with the fossil evidence, firmly placed the origin of *Cycas* in South China, with the deepest divergence explainable by a vicariant event. The RRF represents a large geomorphic discontinuity between the South China and Indochina blocks, which starts from the eastern Himalayas and extends southeastward to the South China Sea [49]. In the Tertiary, it experienced a ca. 325-km left-lateral ductile displacement 27–16 Mya, followed by exhumation and uplift from a depth of 20–25 km, and dextral, predominantly brittle shear activity in the Plio-Quaternary; the ductile movement of the RRF extruded Indochina southeastward relative to South China, and continued along the coast of Vietnam far to the south [67]. The Miocene change of motion was well congruent with the estimated initial divergence time between Clade I and Clade II. Thus, the RRF could thus have acted as a physical barrier that promoted the vicariant divergence within *Cycas*. Support comes from recognized present floristic discontinuities across the RRF, the Kaiyong line [50]. Until now, no *Cycas* taxon of Clade II is distributed to the north of the RRF, the northernmost species (*C. hongheensis*) reaching the fault. However, several species of Clade I (such as *C. chevalieri*, *C. simplicipinna*, and *C. micholitzii*) have spread across the RRF into Indochina, especially along the east side of the Truong Son Mountains, which, to the east along RRF, divides the Thai-Lao Plateau of central Indochina and the lowlands of Vietnam. This cross-boundary distribution could have resulted from a late southward migration during pronounced cooling and glacial periods in the Pleistocene.

In South China and Indochina, cycads generally grow on low-altitude slopes of ridges and cliffs along river valleys. Their fertile seeds are large, heavy and sink in water, and they contain a virulent toxin, cycasine, which precludes water and animal dispersal over a long distance respectively. Thus, in this inland region, seed dispersal of *Cycas* was likely limited to short distances [68–70]. Since the species have a poor overland dispersal capability, the crust deformation, environmental and climatic changes related to orogenic events would likely stimulate their lineage separation and speciation through vicariance events [71]. In contrast, long-distance dispersal may have played an important role in the diversification of species in sect. *Cycas*. They are widely distributed throughout the Indian and western Pacific Ocean, as well as all non-mainland parts of Southeast Asia, extending into Madagascar and neighbouring regions of eastern Africa. In particular, the members of subsect. *Rumphiae* acquired a spongy layer inside the seed [14]. The spongy endocarp provides these large seed with a buoyancy aid in seawater. Alongside, these seeds evolved mechanisms to maintain their viability after prolonged immersion in sea water [68, 69]. This suggests that the spongy endocarp is a key innovation that allowed *Cycas* to occupy a new adaptive zone and promoted diversification.

## Conclusions

Within species of *Cycas*, a high diversity in paralogs of nrDNA ITS copies was revealed. This was attributed to the existence of putative pseudogenes, recombinants, and non-concerted evolution among functional paralogs. Although both pseudogenes and recombinants had lost their usefulness as phylogenetic signals, phylogenetic inference from functional paralogs provided insights into the evolutionary and biogeographic history of *Cycas*. Even though further support is still needed, a scenario can be rebuilt from the resulting phylogenetic patterns, the ancestral distribution reconstruction and the existence of different seed dispersal capabilities suggesting that both vicariant and long-distance transoceanic dispersal events are drivers for the late-Miocene rapid radiations of *Cycas*.

## Supporting Information

S1 Table List of taxa, their section affiliations, distribution ranges and voucher specimen information of *Cycas* samples used in this study. (PDF)

S2 Table Clone and GenBank accession numbers, length and GC content, presence of the 5.8S motif and the free energy of the 5.8S secondary structure of the ITS1, ITS2 and 5.8S regions of *Cycas* species analyzed. Marked in grey are pseudogenes and recombinants (R). ‘V’ indicates the existence of the 14-bp motif in the 5.8S rDNA gene.
(PDF)

## References

[1] Hendricks JG (1987) The Gondwanan *Cycas* . Encephalartos 10: 24–25.

[2] Chamberlain C (1919) The Living Cycads. Chicago: University of Chicago Press.

[3] Loconte H, Stevenson DW (1990) Cladistics of the Spermatophyta. Brittonia 42: 197–211.

[4] Norstog KJ, Nicholls TJ (1997) The Biology of the Cycads. Ithaca, NY: Cornell University Press.

[5] Brenner ED, Stevenson DW, Twigg RW (2003) Cycads: evolutionary innovations and the role of plant—derived neurotoxins. Trends Plant Sci 8: 446–452. 13678912 10.1016/S1360-1385(03)00190-0

[6] Norstog KJ (2003) Foreword In: Donaldson J, editors. Cycads: Status, Survey, and Conservation Action Plan. IUCN/SSC Cycad Specialist Group. IUCN, Gland, Switzerland, and Cambridge, UK pp. 3–8.

[7] Hermsen EJ, Taylor EL, Taylor TN (2009) Morphology and ecology of the *Antarcticycas* plant. Rev Palaeobot Palyno 153: 108–123.

[8] Osborne R (1995) The world cycad census and a proposed revision of the threatened species status for cycad taxa. Biol Conserv 71: 1–12.

[9] Stevenson DW (1990) Morphology and systematics of the Cycadales. Mem N Y Bot Gard 57: 8–55.

[10] Rai HS, O’Brien HE, Reeves PA, Olmstead RG, Graham SW (2003) Inference of higher-order relationships in the cycads from a large chloroplast data set. Mol Phylogenet Evol 29: 350–359. 13678689 10.1016/s1055-7903(03)00131-3

[11] Bogler DJ, Francisco—Ortega J (2004) Molecular systematic studies in cycads: evidence from trnL intron and ITS2 rDNA sequences. Bot Rev 70: 260–273.

[12] Chaw SM, Walters TW, Chang CC, Hu SH, Chen SH (2005) A phylogeny of cycads (Cycadales) inferred from chloroplast matK gene, trnK intron, and nuclear rDNA ITS region. Mol Phylogenet Evol 37: 214–234. 16182153 10.1016/j.ympev.2005.01.006

[13] Osborne RM, Calonje A, Hill KD, Stanberg L, Stevenson DW (2012) The world list of cycads. Mem N Y Bot Gard 106: 480–510.

[14] de Laubenfels DJ, Adema F (1998) A taxonomic revision of the genera *Cycas* and *Epicycas* gen. nov. (Cycadaceae). Blumea 43: 351–400.

[15] Smitinand T (1971) The genus *Cycas* Linn. (Cycadaceae) in Thailand . Nat Hist Bull Siam Soc 24: 163–175.

[16] Hill KD (1995) Infrageneric relationships, phylogeny, and biogeography of the genus *Cycas* (Cycadaceae) In: Vorster P, editors. Proceedings of the Third International Conference of Cycad Biology. Stellenbosch: Cycad Society of South Africa pp. 139–162.

[17] Hill KD (2004) Character evolution, species recognition and classification concepts in the Cycadaceae In: Walters T, Osborne R, editors. Cycad classification, concepts and recommendations. Wallingford: CABI Publishing pp. 23–44.

[18] Wang DY (2000) Studies on morphology, anatomy, taxonomy and evolution of Cycadaceae. PhD Thesis, Nanjing Forestry University, China.

[19] Nagalingum NS, Marshall CR, Quental TB, Rai HS, Little DP, et al (2011) Recent Synchronous Radiation of a Living Fossil. Science 334: 796–799. 10.1126/science.1209926 22021670

[20] Treutlein J, Wink M (2002) Molecular phylogeny of cycads referred from rbcL sequening. Naturwissenschaften 89: 211–215. 10.1007/s00114-002-0308-0 12135087

[21] Crisp MD, Cook LG (2011) Cenozoic extinctions account for the low diversity of extant gymnosperms compared with angiosperms. New Phytol 192: 997–1009. 10.1111/j.1469-8137.2011.03862.x 21895664

[22] Salas-Leiva D, Meerow AW, Calonje M, Griffith P, Francisco-Ortega J, et al (2013) Phylogeny of the cycads based on multiple single copy nuclear genes: congruence of concatenation and species tree inference methods. Ann Bot 112: 1263–1278. 10.1093/aob/mct192 23997230 PMC3806525

[23] Amaral AR, Jackson JA, Möller LM, Beheregaray LB, Coelho MM (2012) Species tree of a recent radiation: the subfamily Delphininae (Cetacea, Mammalia). Mol Phylogenet Evol 64: 243–253. 10.1016/j.ympev.2012.04.004 22503758

[24] Sangin P, Lindstrom AJ, Kokubugata G, Chaiprasongsuk M, Mingmuang M (2010) Phylogenetic relationships within Cycadaceae inferred from non-coding regions of chloroplast DNA. Kasetsart Journal: Natural Scie 44: 544–557.

[25] Xiao LQ, Möller M, Zhu H (2010) High nrDNA ITS polymorphism in the ancient extant seed plant *Cycas*: Incomplete concerted evolution and the origin of pseudogenes. Mol Phylogenet Evol 55: 168–77. 10.1016/j.ympev.2009.11.020 19945537

[26] Buckler ES, Ippolito A, Holtsford TP (1997) The evolution of ribosomal DNA: Divergent paralogous and phylogenetic implications. Genetics 145: 821–832. 9055091 10.1093/genetics/145.3.821 PMC1207866

[27] Baldwin BG, Sanderson MJ, Porter JM, Wojciechowski MF, Campbell CS, et al (1995) The ITS region of nuclear ribosomal DNA: a valuable source of evidence on angiosperm phylogeny. Ann Mo Bot Gard 82: 247–277.

[28] McDade LA (1992) Hybrids and phylogenetic systematics II. The impact of hybrids on cladistic analysis. Evolution 46: 1329–1346. 28569006 10.1111/j.1558-5646.1992.tb01127.x

[29] Petrov DA, Hartl DL (2000) Pseudogene evolution and natural selection for a compact genome. J Hered 3: 221–227. 10833048 10.1093/jhered/91.3.221

[30] Mai JC, Coleman AW (1997) The internal transcribed spacer 2 exhibits a common secondary structure in green algae and flowering plants. J Mol Evol 44: 258–271. 9060392 10.1007/pl00006143

[31] Álvarez I, Wendel JF (2003) Ribosomal ITS sequences and plant phylogenetic inference. Mol Phylogenet Evol 29: 417–434. 14615184 10.1016/s1055-7903(03)00208-2

[32] Zheng XY, Cai DY, Yao LH, Teng YW (2008) Non-concerted ITS evolution, early origin and phylogenetic utility of ITS pseudogenes in *Pyrus* . Mol Phylogenet Evol 48: 892–903. 10.1016/j.ympev.2008.05.039 18577457

[33] Ochieng JW, Henry RJ, Baverstock PR, Steane DA, Shepherd M (2007) Nuclear ribosomal pseudogenes resolve a corroborated monophyly of the eucalypt genus *Corymbia* despite misleading hypotheses at functional ITS paralogs. Mol Phylogenet Evol 44: 752–764. 17570687 10.1016/j.ympev.2007.04.017

[34] Felsenstein J (1985) Confidence limits on phylogenies: an approach using the bootstrap. Evolution 39: 783–791. 28561359 10.1111/j.1558-5646.1985.tb00420.x

[35] Doyle J (1991) DNA protocols for plants—CTAB total DNA isolation In: Hewitt GM, Johnston A, editors. Molecular Techniques in Taxonomy. Berlin: Springer.

[36] Jobes DV, Thien LB (1997) A conserved motif in the 5.8S Ribosomal RNA (rRNA) gene is a useful diagnostic marker for plant internal transcribed spacer (ITS) Sequences. Plant Mol Biol Rep 15: 326–334.

[37] Padidam M, Sawyer S, Fauquet CM (1999) Possible emergence of new geminiviruses by frequent recombination. Virology 265: 218–225. 10600594 10.1006/viro.1999.0056

[38] Simmons MP, Ochoterena H (2000) Gaps as characters in sequence based phylogenetic analyses. Syst Biol 49: 369–381. 12118412

[39] Müller K (2005) SeqState—primer design and sequence statistics for phylogenetic DNA data sets. Appl Bioinformatics 4: 65–69. 16000015 10.2165/00822942-200504010-00008

[40] Nylander JAA (2004) MrModeltest, version 2.2. Program distributed by the author. Uppsala University: Evolutionary Biology Centre.

[41] Stamatakis A (2006) RAxML-VI-HPC: maximum likelihood-based phylogenetic analyses with thousands of taxa and mixed models. Bioinformatics 22: 2688–2690. 16928733 10.1093/bioinformatics/btl446

[42] Buckler ES, Holtsford TP (1996) *Zea* systematics: ribosomal ITS evidence. Mol Biol Evol 13: 612–622. 8882504 10.1093/oxfordjournals.molbev.a025621

[43] Lewis PO, Holder MT, Holsinger KE (2005) Polytomies and Bayesian phylogenetic inference. Syst Biol 54: 241–253. 16012095 10.1080/10635150590924208

[44] Ronquist F, Huelsenbeck JP (2003) MrBayes 3: Bayesian phylogenetic inference under mixed models. Bioinformatics 19: 1572–1574. 12912839 10.1093/bioinformatics/btg180

[45] Yu Y, Harris AJ, He XJ (2014) RASP (Reconstruct Ancestral State in Phylogenies) 3.0. Available at http://mnh.scu.edu.cn/soft/blog/RASP. 10.1016/j.ympev.2015.03.008 25819445

[46] Hill KD (1998) The Cycad Pages. Available: http://plantnet.rbgsyd.gov.au/PlantNet/cycad. Accessed 10 August 2012.

[47] Chiang TY, Schaal BA (2006) Phylogeography of Plants in Taiwan and the Ryukyu Archipelago. Taxon 55: 31–41. 16507522

[48] Holloway NH (1982) North Palawan Bock, Philippines—Its relation to Asian Myainland and role in evolution of South China Sea. Am Assoc Pet Geol Bull 66: 1355–1383.

[49] Leloup PH, Lacassin R, Tapponnier P, Scharer U, Zhong DL, et al (1995) The Ailao Shan—Red River shear zone (Yunnan China), Tertiary transform boundary of Indochina. Tectonophysics 25: 3–84.

[50] Tanaka (1954) Species problem in *Citrus*. Tokyo: Japanese Society for the Promotion of Scence.

[51] Li XW, Li J (1997) The Tanaka-kaiyong Line—An important floristic line for the study of the flora of East Asia. Ann Mo Bot Gard 84: 888–892.

[52] Hall R (2009) Southeast Asia’s changing palaeogeography. Blumea, 54: 148–161.

[53] Wei XX, Wang XQ (2004) Recolonization and radiation in *Larix* (Pinaceae), evidence from nuclear ribosomal DNA paralogs. Mol Ecol 13: 3115–3123. 15367124 10.1111/j.1365-294X.2004.02299.x

[54] Harris DJ, Newman MF, Hollingsworth ML, Möller M, Clark A (2006) The phylogenetic position of *Aulotandra* (Zingiberaceae). Nord J Bot 23: 725–734.

[55] Bergsten J (2005) A review of long-branch attraction. Cladistics 21: 163–193. 10.1111/j.1096-0031.2005.00059.x 34892859

[56] Graham SW, Olmstead RG, Barrett SCH (2002) Rooting phylogenetic trees with distant outgroups: a case study from the commelinoid monocots. Mol. Biol Evol 19: 1769–1781. 12270903 10.1093/oxfordjournals.molbev.a003999

[57] Yang SL, Meerow AW (1996) The *Cycas pectinata* (Cycadaceae) complex: genetic structure and gene flow. Int J Plant Sci 157: 468–483.

[58] Keppel G, Hodgskiss PD, Plunkett GM (2008) Cycads in the insular South—west Pacific: dispersal or vicariance? J Biogeogr 35: 1004–1015.

[59] Griffith MP, Magellan TM, Tomlinson PB (2014) Variation in Leaflet Structure in Cycas (Cycadales: Cycadaceae): Does Anatomy Follow Phylogeny and Geography? Int J Plant Sci 175: 241–255.

[60] Wang WK, Schaal BA, Chiou YM, Murakami N, Ge XJ, et al (2007) Diverse selective modes among orthologs/paralogs of the chalcone synthase (Chs) gene family of Arabidopsis thaliana and its relative A. halleri ssp. gemmifera. Mol Phylogenet Evol 44: 503–520. 17611127 10.1016/j.ympev.2007.05.006

[61] Hill KD (1996b) A taxonomical revision of the genus *Cycas* (Cycadaceae) in Australia . Telopea 7: 1–63.

[62] Wulff EV (1943) An Introduction to historical plant geography. Waltham: Chronic Botanic Company.

[63] Liu YS, Zhou ZY, Li HM (1991) First discovery of *Cycas* fossil pinnae from the Eocene Guchengzi Formation Northeast China. Chinese Sci Bull 22: 1758–1759.

[64] Jones DL (1993) Cycads of the world. Chatswood NSW: Reed Books.

[65] Wolfe JA (1997) Relations of environmental change to angiosperm evolution during the late Cretaceous and Tertiary In: Iwatsuki K, Raven PH, editors. Evolution and diversifi cation of land plants. Tokyo: Springer-Verlag pp. 269–290.

[66] Collinson ME, Fowler K, Boulter MC (1981) Floristic changes indicate a cooling climate in the Eocene of southern England. Nature 291: 315–317.

[67] Golonka J, Krobicki M, Pajak J, Giang NV, Zuchiewicz W (2006) Phanerozoic Palaeogeography of Southeast Asia. Geolines 20: 40–43.

[68] Dehgan B, Yuen CKKH (1983) Seed morphology in relation to dispersal, evolution and propagation of *Cycas* L. Bot Gaz 144: 412–418.

[69] Hill KD (1996a) Cycads in the Pacific In: Keast A, Miller SE, editors. The origin and evolution of Pacific Islands biotas, New Guinea to eastern Polynesia: patterns and processes. Amsterdam: SPB Academic Publishing, 267–274.

[70] Schneider D, Wink M, Sporer F, Lounibos P (2002) Cycads: their evolution, toxins, herbivores and insect pollinators. Naturwissenschaften 89: 281–294. 12216856 10.1007/s00114-002-0330-2

[71] Che J, Zhou WW, Hu JS, Yan F, Papenfuss TJ, et al (2010) Spiny frogs (Paini) illuminate the history of the Himalayan region and Southeast Asia. Proc Natl Acad Sci USA 107: 13765–13770. 10.1073/pnas.1008415107 20643945 PMC2922240

