# Supplementary material for: Nuclear Ribosomal ITS Functional Paralogs Resolve the Phylogenetic Relationships of a Late-Miocene Radiation Cycad Cycas (Cycadaceae)
Source: PLoS One. 2015 Jan 30;10(1):e0117971. doi: 10.1371/journal.pone.0117971 (PMC4311995; doi:10.1371/journal.pone.0117971)
Supplement: S1 Table — (PDF) [file pone.0117971.s001.pdf]

Table S1. List of taxa, their section affiliations, distribution ranges and voucher specimen information of *Cycas* samples used in this study.

Taxa			Distribution Area (Code[a])	Voucher Numbers	Sample Density[b]
Section	Subsection	Species			
Asiorientales	-	*C. taitungensis*	Taiwan (A)	Xiao,06009	2/2
		C. revoluta	Ryukyu Islands (A)	Xiao,06010	
Panzhihuaenses	-	*C. panzhihuaensis*	Sichuan, China (A)	Xiao,06004	1/1
Wadeanae	-	*C. wadei*	Culion Island (A)	Xiao,06018	2/2
		C. curranii	Palawan Island (A)	Xiao,06019	
Stangerioides	Stangerioides	*C. segmentifida*	China, N. Vietnam (A)	Xiao,06012	9/26
		C. guizhouensis	China (A)	Xiao,06015	
		C. balansae	China, N. Vietnam (A)	Xiao,06016	
		C. multipinnata	China, N. Vietnam (A)	Xiao,06017	

		C. debaoensis	China (A)	Xiao, 06014	
		C. bifida	China, N. Vietnam (A)	Xiao,06006	
	Taiwanianae	*C. parvula*	China (A)	Xiao,06002	
		C. ferruginea	China, N. Vietnam (A)	Xiao,06001	
		C. hainanensis	China (A)	Xiao,06011	
Indosinenses	Indosinenses	*C. siamensis*	S.E. Asia (B)	Xiao,06021	5/15
		C. pectinata	S.E. Asia, N.E. India (B)	Xiao,06025	
		C. elonga	Vietnam (B)	Xiao,06026	
		C. tansachana	Thailand (B)	Xiao,06028	
	Hongheses	*C. hongheensis*	China (B)	Xiao,06027	
Cycas	Cycas	*C. circinalis*	India (C)	Xiao,06024	12/61
		C. apoa	New Guinea (C)	Xiao,06029	
		C. nathorstii	Sri Lanka (C)	Xiao,06031	

		C. javana	Indonesia (C)	Xiao,06033	
	Rumphiae	*C. rumphii*	Indonesia-New Guinea (C)	Xiao, 06023	
		C. seemannii	S.W. Pacific islands (C)	Xiao,06034	
		C. thouarsii	E. Africa, Indian Ocean (C)	Xiao,06037	
	Endemicae	*C. media*	Australia (C)	Xiao,06039	
		C. armstrongii	Australia (C)	Xiao,06041	
		C. cairnsiana	Australia (C)	Xiao,06042	
		C. platyphylla	Australia (C)	Xiao, 06035	
		C. campestris	New Guinea (C)	Xiao,06043	

[a] A represents the distribution area of South China, plus Taiwan-Ryukyu Archipelago, and Palawan islands; B represents Indochina; C represents islands of Southeast Asia plus Malay Peninsula, Indian subcontinent, East Africa and North Australia.

[b] numbers before slashes denote the number of species included in the present study; numbers after slashes are

the number of species in each section.
