# Supplementary material for: Nuclear Ribosomal ITS Functional Paralogs Resolve the Phylogenetic Relationships of a Late-Miocene Radiation Cycad Cycas (Cycadaceae)
Source: PLoS One. 2015 Jan 30;10(1):e0117971. doi: 10.1371/journal.pone.0117971 (PMC4311995; doi:10.1371/journal.pone.0117971)
Supplement: S2 Table — Marked in grey are pseudogenes and recombinants (R). ‘V’ indicates the existence of the 14-bp motif in the 5.8S rDNA gene. (PDF) [file pone.0117971.s002.pdf]

Table S2. Clone and GenBank accession numbers, length and GC content, presence of the 5.8S motif and the free energy of the 5.8S secondary structure of the ITS1, ITS2 and 5.8S regions of *Cycas* species analyzed. Marked in grey are pseudogenes and recombinants (R). 'V' indicates the existence of the 14-bp motif in the 5.8S rDNA gene.

		GenBank	Length bp (GC %)			5.8S	5.8SΔG
Taxon	Clone No.	Accessions	ITS1	ITS2	5.8S	Motif	(kcal/mol)
C. pectinata	1	KC508046	671(50.5)	243(55.1)	161(44.1)		-11.9
	4	KC507950	672(49.8)	243(46.5)	161(42.2)		-10.3
	5	KC508077	673(49.2)	243(46.9)	161(42.3)		-14.2
	7R	KC508052	670(52.0)	243(49.8)	161(46.6)	V	-12.6
	13	KC507960	660(64.2)	246(67.1)	161(54.7)		-15.0
	14	KC507955	660(63.7)	246(66.7)	161(55.3)	V	-16.9
	16	KC507954	660(63.7)	246(65.9)	161(54.7)	V	-15.9
	4	KC508057	668(50.8)	243(56.7)	161(46.6)		-10.5

C. multipinnata	5	KC508009	675(64.0)	240(66.7)	161(55.3)	V	-16.9
	10	KC508043	675(55.1)	243(57.2)	160(50.0)	V	-11.7
	13	KC508012	675(64.2)	240(66.7)	161(55.3)	V	-16.9
	14	KC508010	676(63.9)	240(66.7)	161(55.9)	V	-16.7
	16	KC507997	675(64.2)	240(66.3)	161(55.3)	V	-16.9
	18	KC508044	671(55.0)	243(52.3)	161(46.0)	V	-13.2
	1	KC507964	660(63.6)	240(65.8)	161(54.7)	V	-17.1
	5	KC508048	675(50.7)	243(51.9)	161(42.2)		-12.3
C. hongheensis	8	KC507966	674(60.8)	245(61.6)	161(52.8)	V	-20.8
	10	KC508060	671(51.1)	243(49.8)	161(41.6)		-7.8
	11	KC507965	674(63.2)	240(67.1)	161(55.9)	V	-17.1
	3	KC508016	679(64.2)	240(66.7)	161(55.3)	V	-16.9
C. parvula	4	KC508018	675(63.0)	240(64.2)	161(55.3)		-16.9

	5	KC508017	674(63.3)	240(64.6)	161(54.0)	V	-15.6
	1	KC507993	674(64.1)	240(66.7)	161(55.3)	V	-16.9
	2	KC507994	676(64.0)	240(66.7)	161(55.3)	V	-16.9
C. segmentifida	3	KC507998	675(64.2	240(66.7)	161(55.3)	V	-16.9
	4	KC507996	675(64.2)	240(66.7)	161(55.3)	V	-16.9
	5	KC508000	675(64.2)	240(66.7)	161(55.3)	V	-16.9
	1	KC508075	677(50.8)	243(50.6)	161(41.0)		-11.8
	3	KC508062	677(50.1)	233(52.0)	161(42.9)		-8.7
	9	KC508053	651(51.1)	243(51.1）	161(46.5)		-11.9
	12	KC508065	653(50.8)	243(48.9)	161(41.6)		-12.5
	14	KC508025	676(62.3)	244(64.7)	161(54.7)	V	-14.9
C. taitungensis	15	KC508022	676(64.0)	244(65.2)	161(55.9)	V	-16.4
	16R	KC508024	676(63.0)	244(65.1)	161(55.9)	V	-16.4

	17	KC508023	676(64.2)	244(65.2)	161(55.9)	V	-16.4
	18R	KC508041	677(49.9)	243(51.4)	161(43.5)		-13.5
	2	KC508068	679(49.1)	243(49.4)	161(43.5)		-9.0
	3	KC507951	662(48.3)	243(52.3)	161(41.6)		-10.6
C. thouarsii	5	KC507969	662(65.0)	246(64.6)	161(55.3)	V	-16.2
	8R	KC508069	677(49.9)	243(50.2)	161(43.5)		-10.9
	13	KC507968	662(65.1)	246(65.0)	161(55.3)	V	-16.2
	21R	KC508034	673(56.1)	244(57.8)	161(48.5)		-11.9
	2	KC508059	678(50.5)	243(45.7)	161(41.6)		-10.6
	6	KC508058	676(48.5)	243(48.9)	161(41.6)		-8.0
	11	KC507975	664(65.5)	244(64.8)	161(55.3)	V	-16.9
C. campestris	13	KC507977	666(65.1)	244(64.4)	161(55.3)	V	-16.9
	14	KC507976	664(64.6)	244(64.8)	161(55.3)	V	-16.9

	15	KC507974	664(65.3)	244(65.2)	161(55.3)	V	-16.9
	3	KC508032	680(63.3)	244(66.0)	161(52.8)	V	-18.9
C. curranii	8	KC508049	673(49.3)	243(48.6)	161(49.7)	V	-16.3
	9	KC508080	668(44.8)	241(46.5)	160(38.1)		-12.3
	15	KC508029	681(63.4)	244(64.6)	161(55.3)	V	-17.2
	5	KC508036	674(54.6)	243(57.6)	161(50.4)		-15.1
C. armstrongii	12	KC507981	660(63.6)	244(64.8)	161(55.9)	V	-21.9
	15	KC507982	660(64.5)	244(65.2)	161(53.9)		-16.8
	17	KC508070	671(47.4)	243(50.2）	161(43. 5)		-13.8
	1	KC508020	677(64.3)	240(65.0)	161(55.3)	V	-19.0
C. hainanensis	3	KC508072	672(50.3)	243(51.0)	161(42.9)		-9.5
	6	KC508021	677(64.2)	240(65.0)	161(55.3)	V	-19.0
	1	KC508002	675(64.1)	240(66.3)	161(55.9)	V	-16.9

C. balansae	2	KC507995	677(63.3)	240(66.7)	161(54.7)	V	-19.2
	3	KC507999	676(63.7)	240(65.4)	161(54.7)	V	-15.9
	2	KC508013	679(63.7)	240(66.3)	161(55.9)	V	-16.7
C. guizhouensis	3	KC508015	679(64.5)	240(66.3)	161(55.3)	V	-16.9
	4	KC508014	679(64.2)	240(66.3)	161(54.7)	V	-15.9
	3R	KC508037	676(52.9)	246(65.4)	161(55.3)	V	-16.9
C. nathorstii	6	KC507978	660(64.7)	244(65.2)	161(51.6)		-15.6
	11	KC507979	659(65.1)	244(65.2)	161(55.3)	V	-16.9
	12R	KC507967	659(57.0)	242(59.9)	161(53.5)	V	-16.8
	2	KC508079	674(48.5)	243(45.3)	161(42.8)		-13.7
	3	KC508067	641(49.6)	243(48.9)	161(39.8)		-13.1
	7	KC508071	671(49.7)	243(48.2）	161(42.8)		-9.8
C. media	9	KC508035	675(59.2)	243(61.7)	159(48.4)	V	-10.2

	11	KC507987	660(65.0)	244(65.6)	161(55.9)	V	-18.9
	13	KC507985	661(65.2)	244(65.6)	161(55.9)	V	-18.9
	14	KC507986	661(64.9)	244(65.6)	161(55.9)	V	-18.9
	15	KC507983	661(65.1)	244(65.2)	161(55.9)	V	-18.9
	1	KC507952	670(48.9)	243(46.5)	161(43.5)	V	-12.0
	3	KC508073	675(47.3)	219(50.2)	161(45.3)		-10.8
C. apoa	5	KC508076	666(48.8)	243(49.8)	161(36.7)		-6.7
	11	KC507989	660(65.0)	244(66.0)	161(52.8)	V	-17.4
	12	KC507990	660(64.4)	243(62.6)	161(55.3)	V	-16.9
	14	KC507988	660(65.3)	244(66.0)	161(55.3)	V	-16.9
	1	KC508066	670(47.0)	243(51.9)	161(42.3)	V	-10.4
	4	KC508078	675(47.7)	243(49.8)	160(44.4)	V	-12.3
C. tansachana	5	KC507961	660(63.6)	245(66.5)	161(55.3)	V	-16.9

	11	KC507962	660(60.5)	246(62.6)	161(53.4)	V	-15.3
	14	KC507959	660(61.5)	246(67.1)	161(55.3)	V	-16.9
C. cairnsiana	13	KC507992	660(63.3)	244(62.3)	161(51.6)	V	-17.6
	14	KC507984	661(64.3)	244(65.6)	161(55.9)	V	-18.9
	22	KC507980	660(63.8)	240(65.4)	161(55.9)	V	-18.9
	23	KC508050	670(48.7)	243(53.1)	119(42.0)		-7.7
C. javana	5	KC508039	674(53.9)	243(56.4)	157(47.1)	V	-11.1
	6	KC507953	671(49.5)	243(50.6)	161(41.7)		-12.7
	8	KC508061	669(47.9)	199(60.7)	161(42.8)		-9.4
	11	KC507991	660(61.7)	244(60.6)	161(54.0)	V	-12.8
	14	KC507970	660(65.0)	246(65.0)	161(55.3)	V	-16.2
	1	KC508054	675(50.1)	243(52.2)	161(48.5)		-13.2
	3	KC508003	675(63.9)	240(66.7)	161(55.3)	V	-16.9

	4	KC508005	675(63.7)	240(65.5)	161(55.3)	V	-16.9
C. ferruginea	5	KC508064	662(48.4)	243(52.7)	161(42.3)	V	-7.1
	6R	KC508019	671(56.5)	240(66.7)	161(55.3)	V	-16.9
	9	KC508063	673(49.3)	243(48.9)	161(45.3)		-13.3
	12	KC508004	675(64.0)	224(66.7)	161(54.7)	V	-16.9
	13	KC508055	676(50.6)	243(49.4)	161(45.3)		-10.6
	1R	KC508040	672(55.3)	243(53.9)	161(55.9)	V	-16.4
	3	KC508026	681(64.0)	246(65.5)	161 (55.9)	V	-16.4
C. panzhihuaensis	4	KC508027	682(63.8)	246(65.9)	161(55.9)	V	-16.4
	9	KC508047	675(51.6)	243(49.8)	161(44.7)		-11.3
	18	KC508028	680(64.1)	244(66.4)	161(55.9)	V	-16.4
	3	KC507971	660(65.0)	246(65.0)	161(54.7)	V	-14.1
C. seemannii	8	KC507972	661(65.2)	246(65.0)	161(54.7)	V	-14.1

	10	KC507973	661(63.5)	246(65.0)	161(55.3)	V	-16.2
	6	KC508074	681(49.8)	243(49.8)	160(48.1)		-11.5
	7	KC508056	675(47.5)	243(50.2)	161(42.8)		-11.4
C. elonga	14	KC507957	660(63.8)	246(66.7)	161(55.3)	V	-16.9
	15	KC507958	660(64.1)	246(67.1)	161(55.3)	V	-16.9
	16	KC507956	660(63.4)	246(66.7)	161(55.3)	V	-16.9
	18	KC507963	660(62.8)	246(65.5)	161(54.7)	V	-16.9
	7R	KC508038	671(50.5)	244(56.0)	161(55.3)	V	-17.2
C. wadei	13	KC508031	678(62.4)	244(64.8)	161(54.7)	V	-15.0
	15	KC508030	678(63.9)	244(62.8)	161(55.3)	V	-17.2
	16	KC508033	676(58.9)	244(65.2)	161(52.8)	V	-18.6
	2	FJ907972	676(64.4)	244(65.2)	161(55.9)	V	-16.4
	3	FJ908060	674(51.0)	243(46.1)	161(44.7)	V	-13.8

C. revolute	5	FJ907974	676(64.2)	244(65.1)	161(55.9)	V	-16.4
	6	FJ908059	676(49.4)	243(49.4)	161(43.5)		-8.5
	8	FJ908051	671(50.9)	243(50.6)	161(37.2)		-10.9
	11	FJ908056	679(50.5)	242(50.0)	161(42.9)		-9.4
	1	FJ908069	670(49.2)	243(45.7)	160(46.9)		-12.2
C. rumphii	5	FJ908001	635(63.9)	246(63.8)	161(54.1)	V	-15.5
	6	FJ908016	660(64.0)	246(65.0)	161(55.9)	V	-17.8
	10	FJ908015	661(64.0)	246(65.0)	161(55.3)	V	-16.2
	2	FJ908018	657(63.9)	244(65.6)	161(55.7)	V	-17.9
	9	FJ908024	661(64.3)	244(64.4)	161(55.3)	V	-18.1
C. platyphylla	12	FJ908021	661(65.5)	244(65.6)	161(55.3)	V	-18.2
	17	FJ908048	678(49.7)	243(48.6)	161(46.0)		-10.7
	25	FJ908043	671(50.8)	243(50.2)	161(41.0)		-10.0

	8	FJ908014	660(63.8)	246(62.2)	161(54.7)	V	-16.9
C. circinalis	10	FJ908012	660(64.1)	246(63.8)	161(54.7)	V	-17.2
	11	FJ908033	676(53.9)	243(55.6)	161(45.3)	V	-10.4
	15	FJ908008	661(65.3)	246(62.2)	161(55.3)	V	-16.2
	3	FJ907993	659(63.4)	246(66.7)	161(55.3)	V	-16.9
	5	FJ907991	660(63.4)	246(66.7)	161(55.9)	V	-17.2
C. siamensis	10	FJ907990	660(63.5)	246(66.3)	161(55.3)	V	-18.4
	13	FJ908064	675(47.0)	243(48.9)	161(44.1)		-7.3
	15R	FJ907968	671(52.2)	242(50.0)	161(46.6)		-12.2
	2	FJ907984	675(64.0)	240(66.3)	161(55.3)	V	-16.9
	3	FJ907985	674(63.3)	240(66.3)	161(55.3)	V	-16.9
C. debaoensis	5	FJ907970	672(50.0)	243(48.1)	161(45.4)		-11.6
	6	FJ907986	675(64.4)	240(66.3)	161(55.9)	△	-16.9

	15	FJ908068	674(50.4)	243(52.7)	152(44.1)		-10.8
	18	FJ908042	679(52.1)	243(50.2)	161(47.2)		-7.1
	1R	KC508042	680(57.1)	243(53.5)	161(49.7)		-13.3
	2	KC508011	675(60.6)	240(66.3)	161(55.3)	V	-16.9
	3	KC508001	675(64.1)	242(66.1)	161(55.3)	V	-16.9
C. bifida	4	KC508007	675(63.7)	240(66.7)	161(55.3)	V	-16.9
	7	KC508045	676(53.4)	243(48.6)	161(44.7)	V	-10.0
	8	KC508051	671(50.2)	240(66.3)	161(39.1)		-9.2
	11	KC508008	676(64.1)	240(67.1)	161(55.9)	V	-17.1
	22	KC508006	677(64.0)	240(62.2)	161(55.3)	V	-16.9
